# Tandem Mass Tag-based quantitative proteomics analysis of metabolic associated fatty liver disease induced by high fat diet in mice

**DOI:** 10.1186/s12986-020-00522-3

**Published:** 2020-11-18

**Authors:** Hu Li, Wei Huang, Mingjie Wang, Peizhan Chen, Li Chen, Xinxin Zhang

**Affiliations:** 1 Department of Infectious Disease, Research Laboratory of Clinical Virology, Ruijin Hospital, Shanghai Jiaotong University, School of Medicine, Shanghai, 200025 China; 2 Clinical Research Center, Ruijin Hospital, Shanghai Jiao Tong University, School of Medicine, Shanghai, 201821 China; 3 Department of Gastroenterology and Hepatology, Ruijin Hospital, Shanghai Jiao Tong University, School of Medicine, Shanghai, 201821 China

**Keywords:** Metabolic associated fatty liver disease, Proteomics, Tandem Mass Tag

## Abstract

**Background:**

Although metabolic associated fatty liver disease (MAFLD) is the most common chronic liver disease worldwide, the exact molecular mechanism of MAFLD progression remains unknown. In the present study, Tandem Mass Tag-labeled quantitative proteomic technology was used to elucidate the protein expression patterns of liver tissues in the progression of MAFLD, providing new potential therapeutic targets of it.

**Methods:**

Five 6-week-old male C57BL/6 mice were fed with high fat diet (HFD) for 22 weeks to establish the MAFLD mouse models. Five C57BL/6 mice of the same age were fed with normal diet (ND) and taken as controls. Mice serum were sampled for biochemical tests, and livers were isolated for histopathological examinations. Six mouse liver samples (three from each group) were performed for proteomic analysis. Differentially expressed proteins were defined using fold change of > 1.5 or < 0.67 and *p* value < 0.05 as thresholds. Bioinformatic analysis was used to identify the hub proteins. Real-Time Quantitative Polymerase Chain Reaction (RT-qPCR), Gene Expression Omnibus dataset, western blotting and immunohistochemistry were used to validate the expression of identified hub proteins.

**Results:**

After 22 weeks on HFD diet, all mice developed MAFLD demonstrated by histopathological examination. Mouse body weights, liver weights, serum alanine transaminase and aspartate transaminase levels were significantly higher in the HFD group than ND group. Proteomics technology identified 4915 proteins in the mouse livers, among which 71 proteins were differentially expressed. Kyoto Encyclopedia of Genes and Genomes pathway analysis showed that majority of the differentially expressed proteins were involved in the peroxisome and peroxisome proliferator-activated receptor signaling pathway, as well as biosynthesis of unsaturated fatty acids. Protein–protein interaction analysis showed that these differentially expressed proteins interacted with each other and formed a complex network. Ten hub proteins were identified and validated using RT-qPCR. Five of these proteins were validated in the Gene Expression Omnibus dataset. Finally, Enoyl-CoA hydratase and 3-hydroxyacyl CoA dehydrogenase protein was validated in mouse liver tissue samples using western blotting and immunohistochemistry.

**Conclusion:**

Our data showed that lipid metabolism-related pathways are closely associated with the development of MAFLD. The identified hub proteins might be novel targets for treating MAFLD.

## Introduction

Metabolic associated fatty liver disease (MAFLD), formerly named non-alcoholic fatty liver disease (NAFLD) [1], is a rapidly growing metabolic disease associated with type-2 diabetes mellitus and obesity; it is currently the most common chronic liver disease worldwide [2]. Epidemiological studies and prediction models show that the overall prevalence of MAFLD in the general population is about 25–30% [2, 3]; it is projected to increase to 33.5% by 2030 [4]. The prevalence of this disease could even increase to above 50% in the global diabetes population [5]. MAFLD is a heterogeneous disease comprising of simple fatty liver, metabolic associated steatohepatitis (MASH), previously known as non-alcoholic steatohepatitis (NASH), and fatty liver related cirrhosis. MASH, the later stage of MAFLD [6] is characterized by a varied degree of hepatocellular ballooning, lobular inflammation and liver fibrosis, which is more likely to progress into liver cirrhosis, and even hepatocellular carcinoma [7].

The pathogenesis of MAFLD is complex, and the exact mechanism of MAFLD development is still largely unknown. The traditional ‘two-hit’ hypothesis holds that a ‘second-hit’ (oxidant stress, etc.) promotes the progression of MAFLD in the setting of hepatic steatosis as the ‘first-hit’ [8]. However, in the recent years, it has been generally accepted that the onset and progression of MAFLD are a result of multiple factors, including genetic susceptibility, diet, insulin resistance, and intestinal microbiota disorders [9]. Currently, the main treatments for MAFLD are weight loss and physical exercise, with no effective drugs yet approved for treating MASH [10]. Therefore, a better understanding of the pathogenesis of MAFLD will help find novel therapies for it.

Over the recent years, high-throughput quantitative proteomics have been widely applied to analyze protein expression patterns and identify novel molecular markers for diseases. Proteomics aims to study the composition of proteins in cells, tissues, and organisms, and their dynamic changing laws. Potential novel protein molecules associated with diseases can be identified by comparing the protein expression profiles between healthy individuals and patients; such a comparison provides new insights into the molecular mechanism of disease progression and allows for the identification of new targets for drug designing [11, 12]. An in vitro study carried out by Xia et al. [13] using iTRAQ-labeled proteomics technology found that heat shock protein 27 (SHP27) plays a key role in the free fatty acid induced steatosis cell model in LO_2_ cell line; they also identified that targeting SHP27 exhibited a protective effect on hepatocyte injury. Yuan et al. [14] compared the protein expression profiles between individuals with metabolically healthy obesity and MAFLD to identify 216 differentially expressed proteins (DEPs); of these, fibulin-5 and short-chain dehydrogenase/reductase family member 2 (DHRS2) protein were validated using western blotting and immunohistochemistry in the study. Nevertheless, limited proteomics studies have been carried out on MAFLD.

In the present study, Tandem Mass Tag (TMT)-based quantitative proteomics technology was employed to identify DEPs that might be involved in MAFLD progression. The potential functions of DEPs were then analyzed, followed by the construction of an interaction network of these proteins. Finally, ten hub proteins, interacting with a larger number of other proteins in a network and playing a key role in the development of diseases, were identified, which were Enoyl-CoA hydratase and 3-hydroxyacyl CoA dehydrogenase (EHHADH), Hydroxysteroid 17-beta dehydrogenase 4 (HSD17B4), Acyl-CoA oxidase 1 (ACOX1), Acetyl-Coenzyme A acyltransferase 1B (ACAA1B), Carnitine O-acetyltransferase (CRAT), Acyl-CoA thioesterase 3 (ACOT3), Acyl-CoA thioesterase 4 (ACOT4), Peroxisomal biogenesis factor 11 alpha (PEX11A), ATP binding cassette subfamily D member 3 (ABCD3), and cytochrome P450, family 2, subfamily b, polypeptide 9 (CYP2B9). These proteins were validated using Real-Time Quantitative Polymerase Chain Reaction (RT-qPCR) and Gene Expression Omnibus (GEO) dataset. EHHADH protein was further validated using western blotting and immunohistochemistry. This study will provide valuable insights into the pathogenesis of MAFLD at the protein level and identify novel targets for the treatment of it.

## Material and methods

### Animal experiments

4-week-old male C57BL/6 mice were purchased from Shanghai SLAC Laboratory Animal Co. Ltd. (Shanghai, China) and housed in specific pathogen free conditions on a 12 h light/dark cycle with free access to water and food. Following acclimatization to the housing environment for 2 weeks, the mice were fed with high fat diet (n = 5, HFD group, D12492, Research Diet, New Brunswick, USA, containing 60% kcal fat, 20% kcal carbohydrate, and 20% protein) for 22 weeks to establish MAFLD mouse models. The control mice were fed with standard normal diet (n = 5, ND group) for 22 weeks. Body weights of mice from both groups were recorded every week. At the end of the study, the mice were anesthetized by intraperitoneally injecting 1% pentobarbital sodium solution at a dose of 50 mg/g. The mice were subsequently euthanized by exsanguination after overnight fasting. Liver samples were collected and stored in liquid nitrogen for histological evaluation and TMT-based proteomics analysis. All animal experiments were performed with approval from the Institutional Animal Care and Use Committee of Ruijin Hospital, Shanghai Jiaotong University School of Medicine.

### Liver histological evaluation

Liver tissues were fixed in 4% neutral-buffered formalin, embedded in paraffin, and then subjected to hematoxylin and eosin (H&E) staining for assessment of liver histology. MAFLD activity score (NAS) was calculated by a sum of scores of steatosis, lobular inflammation and hepatocyte ballooning, as described previously [15]. Oil Red O staining was performed on the frozen sections to evaluate steatosis in the liver lobes using standard methods [16]. A blind evaluation of the liver histology was carried out by two experienced pathologists.

### Sample preparation and TMT labeling

The total proteins were extracted from perfused liver tissues as described previously [17]. Briefly, the perfused liver tissues were grinded into cell powder using liquid nitrogen. The samples were sonicated in four volumes of lysis buffer (8 M urea, 1% Protease Inhibitor Cocktail and 2 mM Ethylene Diamine Tetraacetic Acid) three times on ice using a high intensity ultrasonic processor. After centrifugation at 12,000*g* for 10 min at 4 °C, the supernatant was collected and the protein concentrations were determined using a BCA kit (Beyotime Biotechnology, Shanghai, China), according to the manufacturer`s instructions. 150 μg of protein samples were reduced using 5 mM dithiothreitol for 30 min at 56 °C and alkylated using 11 mM iodoacetamide for 15 min at room temperature followed by dilution with Tetraethylammonium bromide (TEAB). The first digestion was carried out overnight using trypsin at a trypsin-to-protein mass ratio of 1:50, followed by a second digestion for 4 h at a trypsin-to-protein mass ratio of 1:100. Finally, the peptide was desalted using the Strata X C18 SPE column (Phenomenex, California, USA), vacuum-dried, reconstituted in 0.5 M TEAB, and processed using the TMT kit (TMT 10 plex™ Isobaric Label Reagent Set, Thermo Fisher Scientific, USA), according to the manufacturer’s protocol.

### HPLC fractionation, LC–MS/MS analysis, database search, and bioinformatics analysis

The tryptic peptides were separated into 60 fractions by high pH reverse-phase High Performance Liquid Chromatography (HPLC) using the 300Extend C18 column (5 μm particles, 4.6 mm ID, 250 mm length; Agilent, California, USA) with a gradient of 8–32% acetonitrile (pH 9.0) over 60 min, then combined into 9 fractions and dried by vacuum centrifuging. Afterward, the peptides were analyzed using LC–MS/MS on QExactive™ Plus (Thermo Fish Scientific) coupled to an EASY-nLC 1000 UPLC system (Thermo Fisher Scientific). Intact peptides were detected at a resolution of 70,000 and then selected for MS/MS with an NCE setting of 28. The fragments were detected at a resolution of 17,500. MaxQuant search engine (v.1.5.2.8) was used to process the MS/MS data. Tandem mass spectra were searched against SWISS-PROT database (mouse) concatenated with reverse decoy database. Trypsin/P was used as the cleavage enzyme allowing up to two missing cleavages. The mass tolerance for precursor ions in First search and in Main search was set as 20 ppm and 5 ppm, respectively, and for fragment ions was set as 0.02 Da. FDR was set < 1% and minimum score for peptides was set at > 40. Gene ontology (GO) annotation was performed using Uniprot-GOA database (https://www.ebi.ac.uk/GOA/). DEPs were classified by GO annotation based on the following three categories: cellular component (CC), molecular function (MF), and biological process (BP). Kyoto Encyclopedia of Genes and Genomes (KEGG) database (https://www.kegg.jp) was used to identify the protein pathways. WoLF PSORT, a subcellular localization prediction software, was used to predict the subcellular localization [18].

### RNA extraction and RT-qPCR

Total RNA was extracted from mouse livers using TRIZOL reagent (Beyotime Biotechnology). 1 μg of total RNA was reverse-transcribed to cDNA using the PrimeScript™ RT Master Mix (Takara Bio, Japan). RT-qPCR was performed using the QuantNova SYBR Green PCR kit (QIAGEN, Germany) on the Light Cycler® 96 real-time PCR system (Roche, Switzerland). The primers are listed in Additional file 1. The relative expression of mRNA was calculated using the 2−ΔΔCt method.

### Verification of proteins by western blot

About 100 mg of liver tissues were homogenized in 1 ml of Radio-Immunoprecipitation Assay lysis buffer (P0013B, Beyotime Biotechnology, China) and phenylmethanesulfonyl fluoride (ST506, Beyotime Biotechnology, China) with an automatic grinder (JXFSTPRP024, Shanghai Jingxin Industrial development Co., Ltd, China), and the lysates were centrifuged at 14,000 rpm for 15 min at 4 °C. The supernatant was collected. The total protein concentration in the supernatant was quantified using a BCA protein assay kit (Beyotime Biotechnology, China). The total protein (20 μg/sample) was separated with on 10% sodium dodecylsulfate-polyacrylamide gel electrophoresis (SDS-PAGE), and then transferred to polyvinylidene difluoride (PVDF) membranes. The membranes were blocked with 5% milk in PBS with 0.1% Tween-20 (PBST) for 2 h, and then incubated with specific primary antibodies against EHHADH (sc-393123, Santa cruz Biotechnology) overnight at 4 °C, followed by incubation with a horseradish peroxidase-conjugated secondary antibody for 1 h. GADPH protein (AF0006, Beyotime Biotechnology) was used as internal control. Protein bands were visualized using enhanced chemiluminescence (ECL) kit (Tanon Technology Co., Ltd, Shanghai, China).

### Immunohistochemistry analysis

Formalin-fixed liver tissue samples were performed with immunohistochemistry analysis. Briefly, mouse liver sections were deparaffinized, repaired with 0.01 M sodium citrate-hydrochloric acid buffer solution, and incubated with rabbit anti-EHHADH primary antibody (sc-393123, Santa Cruz Biotechnology, 1:200 dilution) at 4 °C overnight. The incubated liver sections were treated with peroxidase-conjugated secondary antibody (111-035-003, Jackson ImmunoResearch, USA) at room temperature, stained with diaminobenzidine for 3–5 min, and counterstained with hematoxylin. The liver sections were then observed under a light microscope.

### Statistical analysis

Data are expressed as mean ± standard deviation. Two-tailed student’s *t* test was used to analyze the differences between two groups. *p* value less than 0.05 was considered statistically significant. Statistical analysis was performed using Graph Pad Prism 8 (version 8.2.1).

## Results

### Histopathological evaluation of livers from mice fed with high fat diet or normal diet

**Fig. 1:**
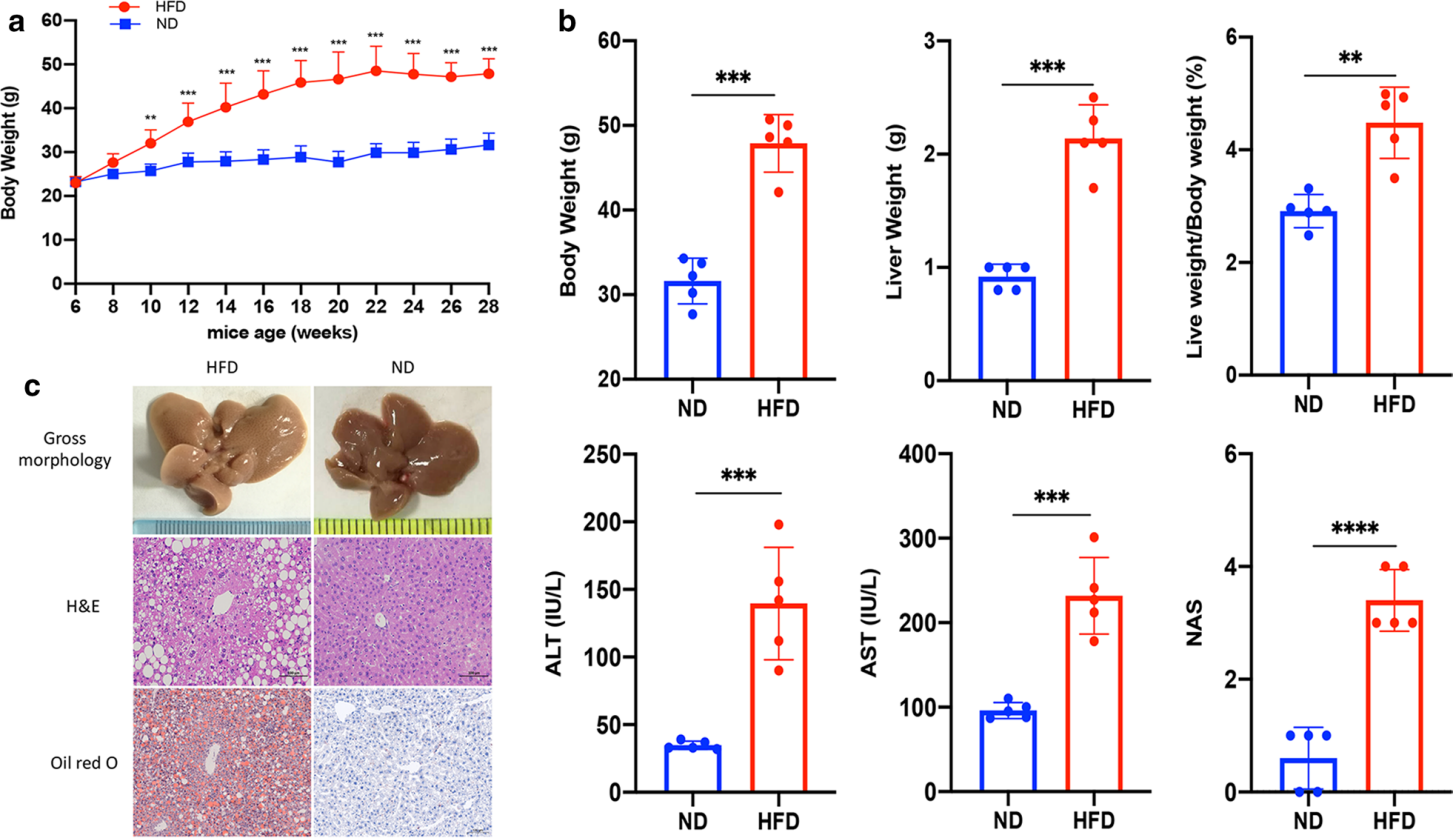
HFD promotes the development of MAFLD. **a** Body weight gain of mice fed with HFD or ND. **b** Mouse body weights, liver weights, liver/body ratios, serum liver tests, and NAS scores of the HFD and ND groups, measured in the 22th week. **c** Gross morphology, H&E and Oil red O staining of livers from HFD- or ND-fed mice. *p < 0.05, **p < 0.01, ***p < 0.001, ****p < 0.000

Mice in the HFD group showed a faster body weight gain compared to the control group; there was a significant difference (32.0 ± 3.1 g vs. 25.7 ± 1.5 g, *p* = 0.003) in the body weights between the two groups from the 10th week onwards (Fig. 1a). After 22 weeks on HFD diet, all mice developed MAFLD. The mean body weight of the HFD-fed mice was 47.3 ± 3.5 g, which was significantly higher than that of the controls (31.6 ± 2.4 g, *p* < 0.001). Moreover, HFD diet also led to a significant increase in liver weight. Mouse liver weights and liver/body ratios were significantly higher in the HFD group than the control group (0.9 ± 0.11 g vs. 2.1 ± 0.29 g, *p* < 0.001 and 3.0 ± 0.27% vs. 4.4 ± 0.69%, *p* = 0.004, respectively). As expected, HFD also elevated the serum alanine transaminase and aspartate transaminase levels compared with ND (*p* < 0.001). The NAS scores of the HFD group were higher than those of the control group [1(0–1) vs. 3(3–4), *p* < 0.001] (Fig. 1b). H&E staining showed that the livers of mice fed with HFD, but not of control mice, were characterized by significant macro-vesicular steatosis of hepatocytes, accompanied by slight ballooning degeneration and lobular inflammation. Oil red O staining confirmed the accumulation of fat drop in the hepatocytes in the HFD group (Fig. 1c).

### Identification and quantification of differentially expressed proteins related to MAFLD

**Fig. 2:**
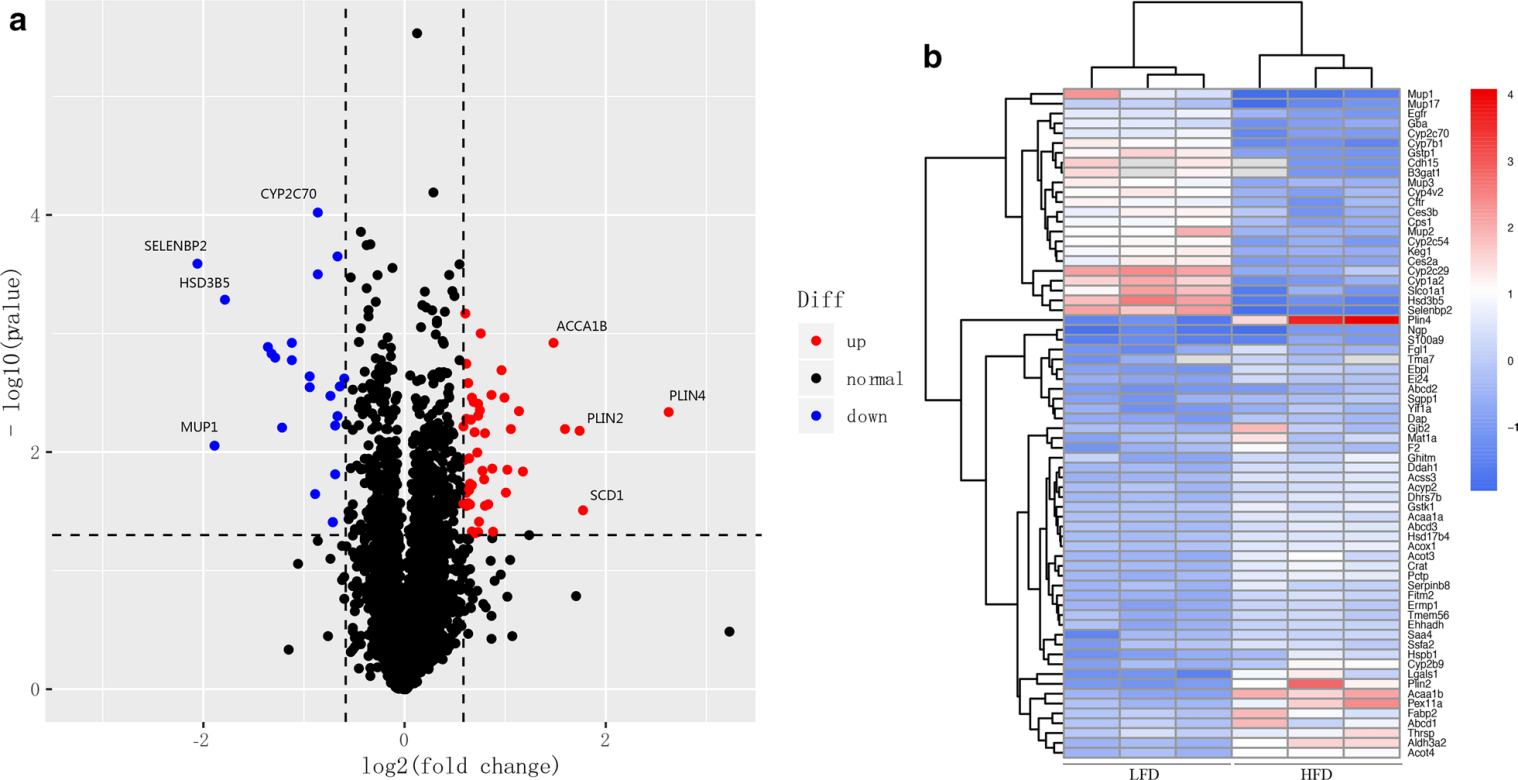
Differentially expressed proteins between HFD and ND groups. **a** Volcano plot showing the DEPs. Red and green dots represent up-regulated and down-regulated proteins, respectively, while black dots represent proteins that did not change significantly. **b** Heat map showing DEPs. The expression values of proteins were normalized with z-scores. Blue and red colors indicate low and high expression of proteins, respectively

A total of 4915 proteins were identified with at least one unique peptide at a false discovery rate of < 1%, of which 83.4% (4101/4915) were quantified on TMT ion channels; About 50% (2291/4101) of these proteins were quantified by more than five peptides. Detailed information about the identified proteins, including protein accession, protein description, Mascot score, coverage, and number of matched peptides, have been listed in Additional file 2. When the proteins with *p* value < 0.05 were considered as DEPs, a total of 666 proteins were identified, among which 349 proteins were up-regulated and 317 proteins down-regulated. Upon using fold change of > 1.5 or < 0.67 and *p* value < 0.05 as thresholds to define DEPs, 71 proteins were identified, among which 48 proteins were up-regulated, while 23 proteins were down-regulated in the HFD-fed mice, compared to the controls (Fig. 2a). The protein expression values of the 71 DEPs normalized with z-scores have been shown in a heatmap (Fig. 2b).

### Subcellular localization, GO and KEGG pathway enrichment analysis of differentially expressed proteins

**Fig. 3:**
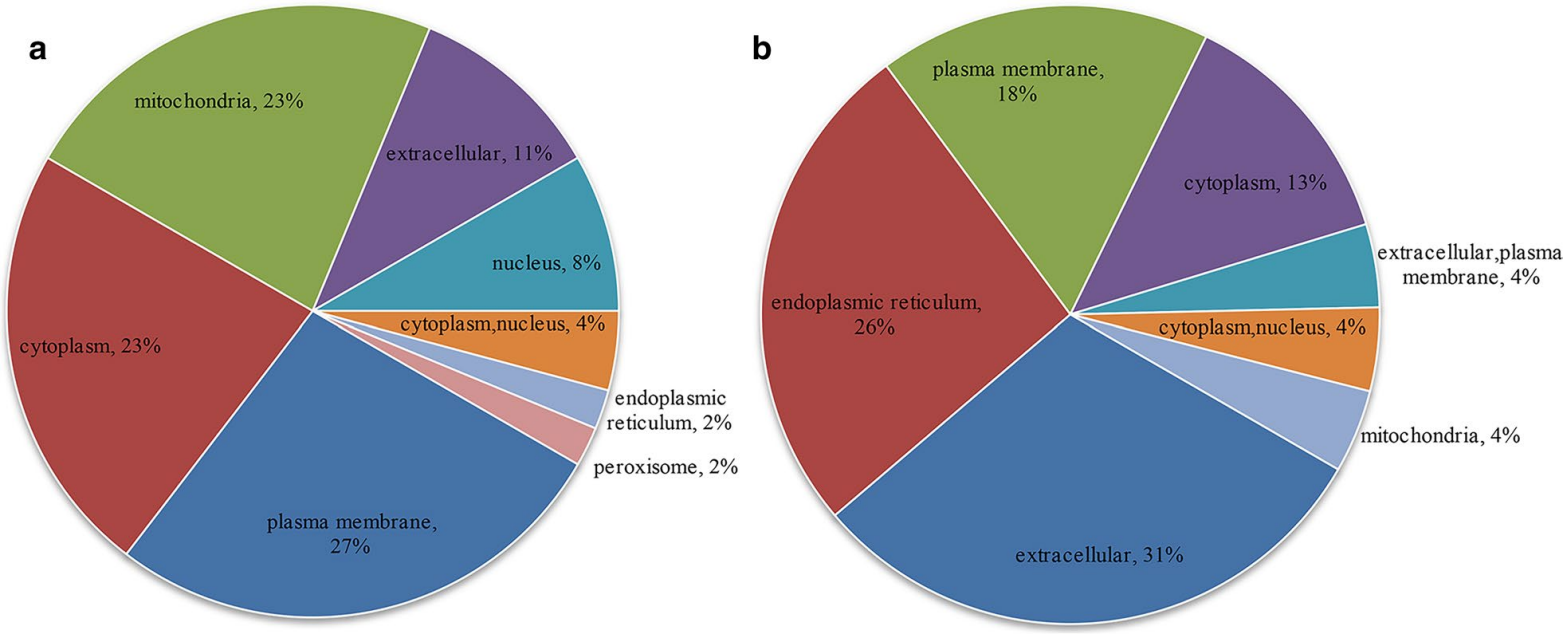
Subcellular localization of 71 differentially expressed proteins. **a** Subcellular localization of upregulated DEPs. **b** Subcellular localization of downregulated DEPs

**Fig. 4:**
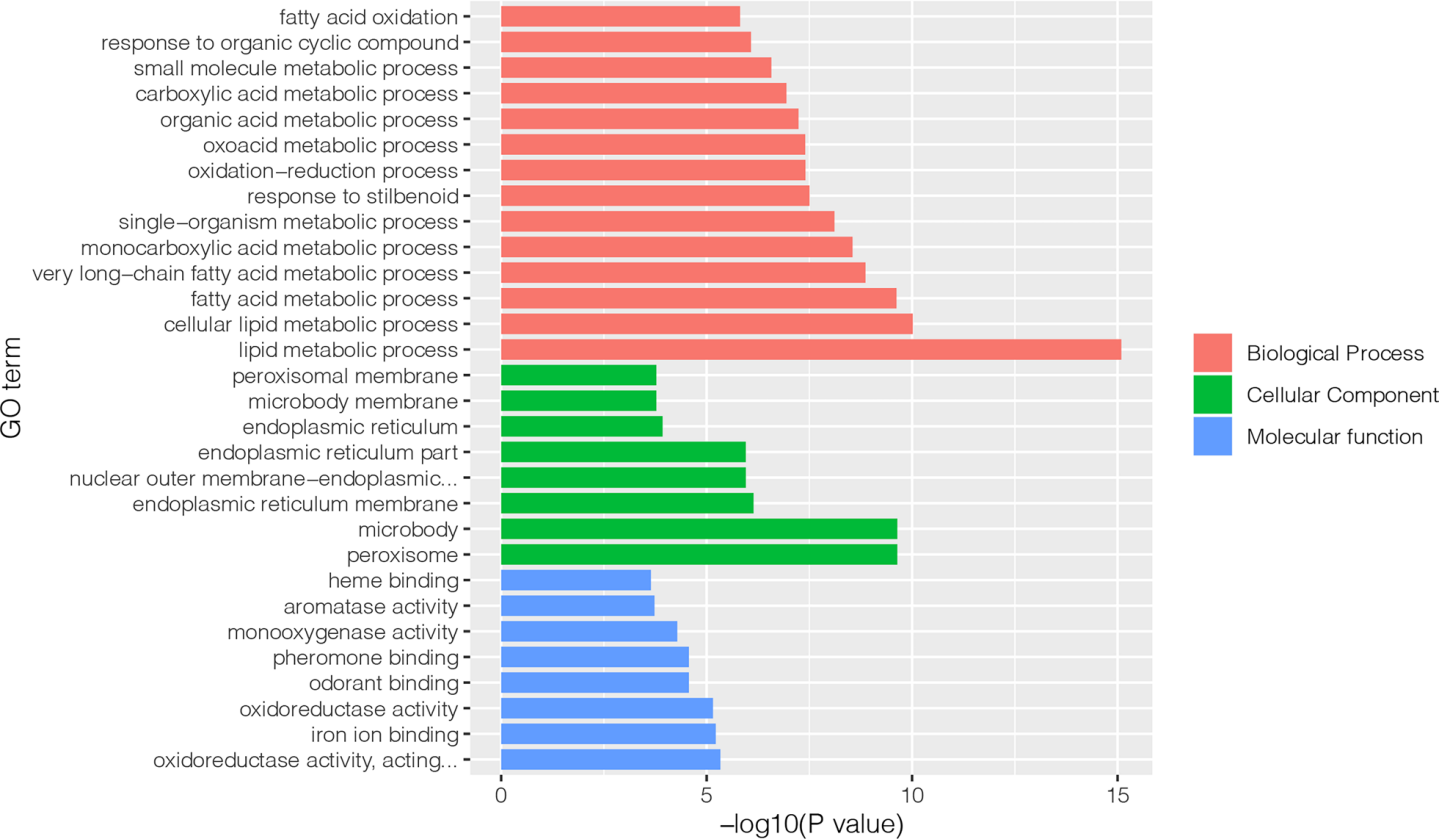
GO analysis of 71 differentially expressed proteins. DEPs are classified into biological process, cellular component and molecular function

**Fig. 5:**
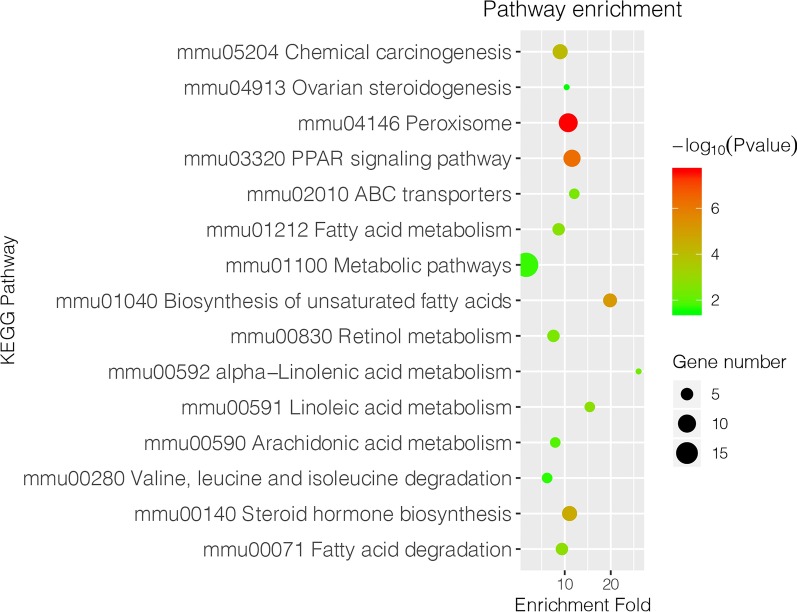
KEGG pathway enrichment analysis of 71 differentially expressed proteins. Size of circles indicates the gene number. Color of the circles represents − log10(*p* value)

71 DEPs with fold change of > 1.5 or < 0.67 and *p* value < 0.05 identified above were subjected to further bioinformatic analysis. Up-regulated proteins were mainly localized in the plasma membrane (27%), cytoplasm (23%), and mitochondria (23%), while down-regulated proteins were mainly localized extracellularly (31%) or in the endoplasmic reticulum (26%) and plasma membrane (18%) (Fig. 3a, b). GO annotation analysis showed that most of the enriched proteins were related to peroxisome, microbody, and endoplasmic reticulum membrane in the CC category. In the BP category, the highly enriched proteins were associated with lipid metabolic process, cellular lipid metabolic process, fatty acid metabolic process and very long-chain fatty acid metabolic process. In the MF category, the enriched proteins were involved in oxidoreductase activity, iron ion binding, odorant binding and pheromone binding (Fig. 4). The results of KEGG pathway analysis indicated that majority of the DEPs were involved in several lipid metabolism-related pathways, including peroxisome, PPAR signaling pathway, biosynthesis of unsaturated fatty acids, and some other pathways (Fig. 5). We then performed GO and KEGG pathway analysis with 666 proteins with *p* value < 0.05. The results of GO analysis showed that lipid metabolism associated processes, such as lipid metabolic process, fatty acid metabolic process, etc., were enriched in the BP category. Moreover, lipid metabolism-related pathways, such as biosynthesis of unsaturated fatty acids, PPAR signaling pathway, were also enriched in KEGG pathway analysis. The results of GO and KEGG pathway analysis with 666 proteins with *p* value < 0.05 are shown in Additional file 3 in more detail.

### Protein–protein interactions network analysis of differentially expressed proteins and identification of hub proteins

**Fig. 6:**
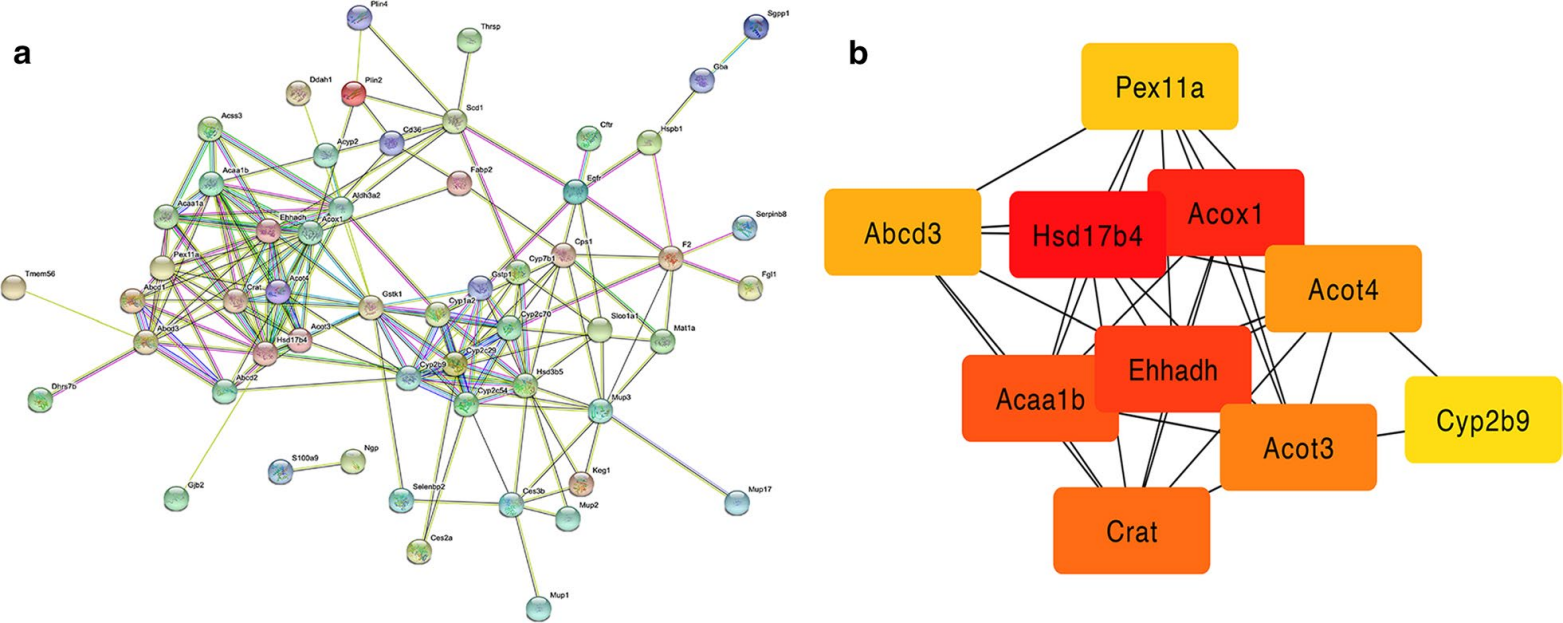
Protein–protein interaction network of 71 differentially expressed proteins and hub proteins. **a** Protein–protein interaction network of DEPs. **b** Protein–protein interaction network of identified hub proteins. Tangerine color represents proteins ranked higher, while yellow color represents proteins ranked lower. Lines between two nodes represent proteins interactions

A PPI network was constructed using the online functional protein association networks database STRING (version 11.0, https://string-db.org/) [19]. Active interaction sources including ‘Textmining’, ‘Experiments’, ‘Databases’, ‘Co-expression’, ‘Neighborhood’ and ‘Co-occurrence’ were selected. 71 DEPs identified above were performed for PPI network analysis with the minimum required interaction score set as 0.4. As a result, the constructed network included a total of 71 nodes and 168 edges with an average node degree 4.73 and a PPI enrichment *p* value below 0.001 (Fig. 6a). MCC algorithm of CytoHubba [20] plug-in in Cytoscape 3.7.2 [21] was used to identify hub proteins from the network. Finally, top 10 proteins (EHHADH, HSD17B4, ACOX1, ACAA1B, CRAT, ACOT3, ACOT4, PEX11A, ABCD3, and CYP2B9) ranked in terms of the MCC algorithm were considered as hub proteins (Fig. 6b). We then performed PPI network analysis using the 666 proteins with *p* value < 0.05 and the minimum required interaction score was set as 0.7, the constructed network had 663 nodes and 1818 edges with PPI enrichment *p* value < 1.0 × 10^–16^ (Additional file 4). Further analysis using cytoscape plug-in CytoHubba identified top 20 hub proteins, including EHHADH, HSD17B4, ACOX1, CRAT, ACOT3 and ACOT4 (Additional file 5).

### Validation of identified differentially expressed proteins using RT-qPCR

**Fig. 7:**
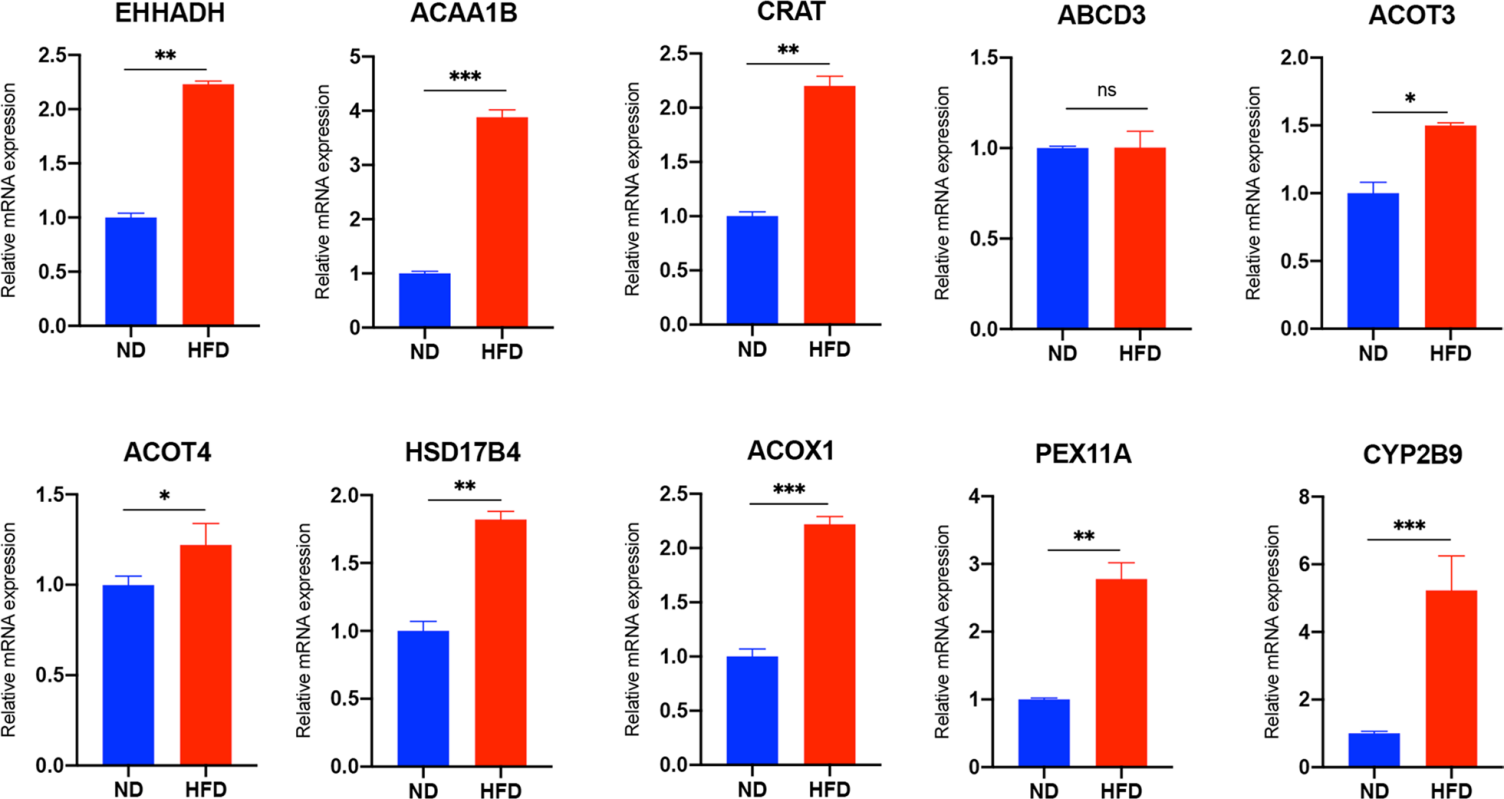
Validation of identified ten hub proteins using RT-qPCR. *HFD* high-fat diet group, *ND* normal diet group. *p < 0.05, **p < 0.01, ***p < 0.001

The expression levels of identified ten hub proteins with fold change of > 1.5 or < 0.67 and *p* value < 0.05 were validated at the transcriptional level using RT-qPCR analysis. In the TMT-based proteomic analysis, all these proteins were up-regulated in the HFD group compared to ND. At the transcriptional level, RT-qPCR analysis results showed that nine of the genes encoding for the hub proteins identified above were significantly up-regulated in the HFD group as compared to the ND group (*p* < 0.05), consistent with the results of TMT-based proteomics results. However, one gene (encoding for ABCD3), found to be up-regulated in the HFD group in the proteomics analysis, did not show significant differences between the two groups when analyzed using RT-qPCR (Fig. 7). The gene primers used for the RT-qPCR analysis are listed in Additional file 1.

### Validation of the hub proteins in biopsy-proven MAFLD patients

**Fig. 8:**
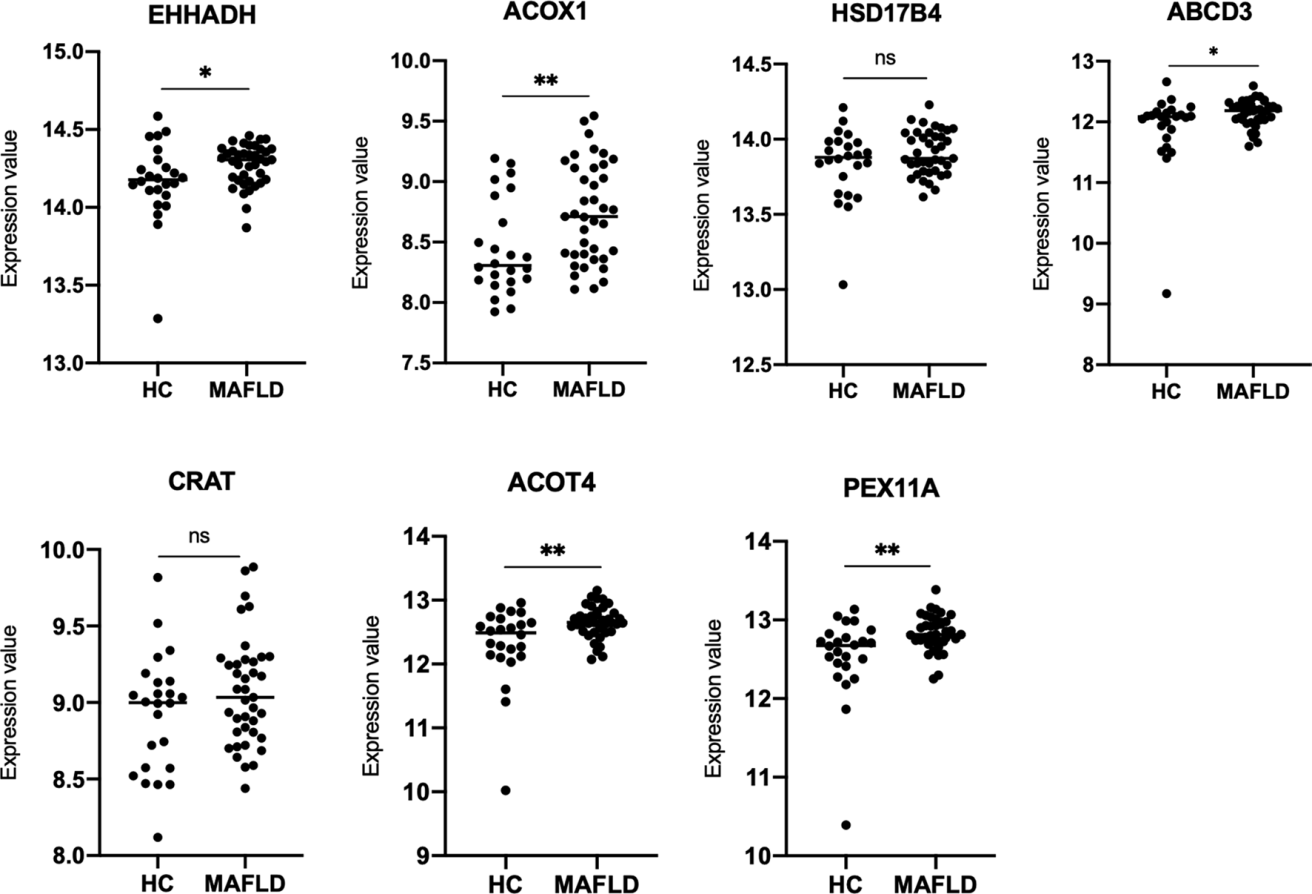
Expression levels of ten hub proteins included in the GEO dataset. *HC* healthy control, *MAFLD* metabolic associated fatty liver disease. *p < 0.05, **p < 0.01, ***p < 0.001

The online GEO microarray expression profiling dataset GSE89632 was used to further validate the hub proteins identified in the TMT-based proteomic analysis. GSE89632 dataset contains data from 24 healthy controls and 39 biopsy-proven MAFLD patients. Seven genes (*EHHADH*, *ACOX1*, *HSD17B4*, *ABCD3*, *CRAT*, *ACOT4* and *PEX11A*) were found to be included in the GSE89632 dataset. Further analysis showed that the expression values of *EHHADH*, *ACOX1*, *ABCD3 ACOT4* and *PEX11A* genes were significantly up-regulated in the MAFLD patients compared to the healthy control (*p* < 0.05), consistent with the proteomics and RT-qPCR results from mouse models in the present study. The expression values of *HSD17B4* and *CRAT* genes, on the other hand, were not significantly different between MAFLD patients and healthy controls (Fig. 8).

### Assessment of EHHADH using western blotting and immunohistochemistry

**Fig. 9:**
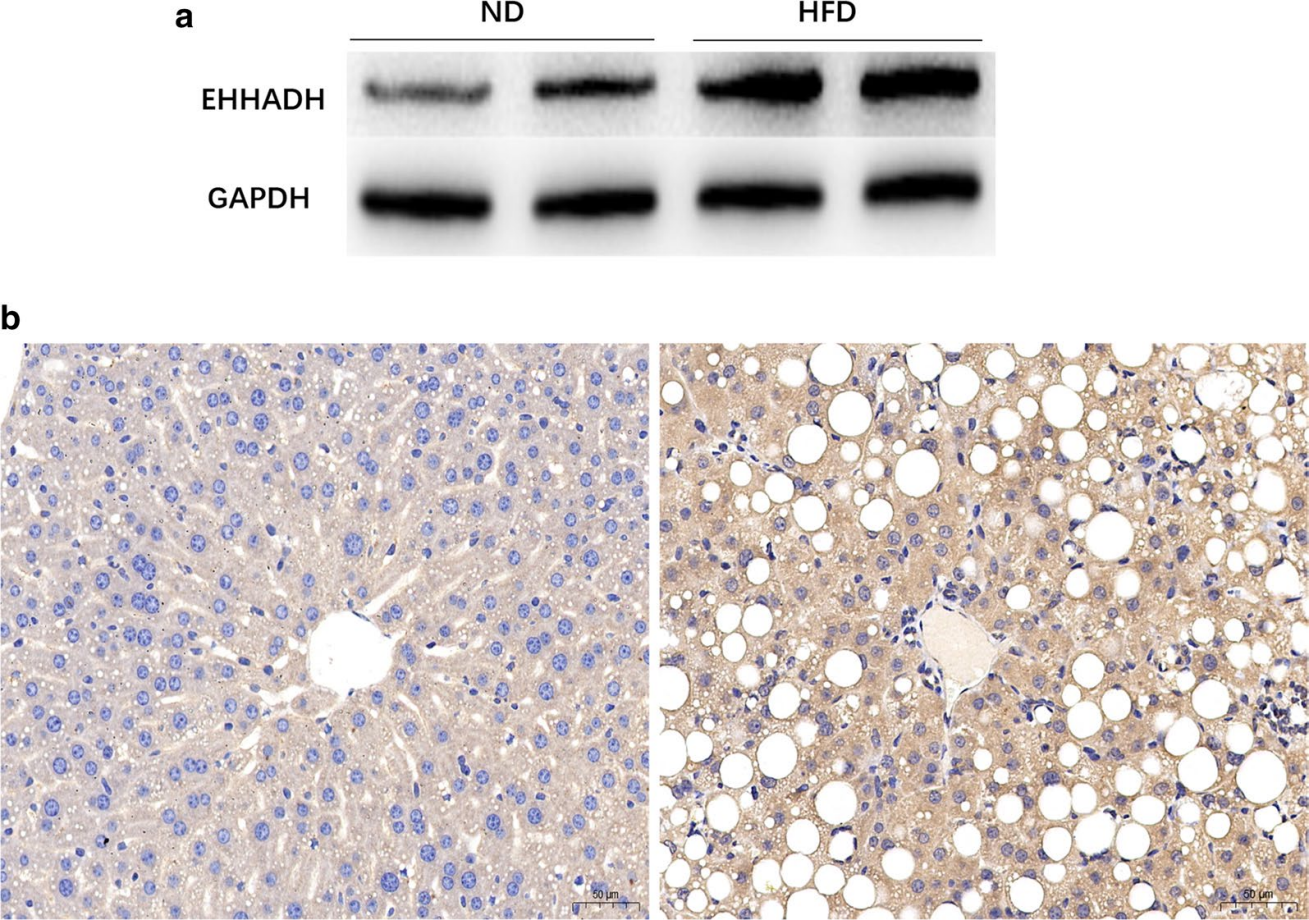
Verification of expression of EHHADH protein expression using western blotting (**a**) and immunohistochemistry (**b**)

Based on the bioinformatics analysis, EHHADH protein was one of the top identified hub proteins, a result confirmed using RT-qPCR and GEO dataset analyses. We decided to further validate EHHADH in the mouse liver samples using western blotting and immunohistochemistry. Both western blotting and immunohistochemistry results confirmed a significant up-regulation of EHHADH in HFD-fed mice at the protein level compared to the controls, thus validating the results of the TMT-based proteomics (Fig. 9a, b).

## Discussion

The present study investigated the proteomic patterns in liver tissues from mice with HFD-induced MAFLD using TMT-based quantitative proteomics technology to elucidate the molecular mechanism of MAFLD progression. We identified and quantified 71 liver tissue proteins that were differentially expressed between the HFD and ND groups. Further bioinformatics analysis revealed that these proteins were involved in many metabolic processes. Ten hub proteins were identified from the PPI network, which were further validated using RT-qPCR. Finally, the most promising of the hub proteins, EHHADH was validated using western blotting and immunohistochemistry.

A recent study comparing the protein expression patterns between subjects with metabolic healthy obesity and MAFLD found that the PPAR signaling pathway was the top up-regulated pathway in the KEGG pathway analysis [14], consistent with our results. PPARs are a subfamily of ligand-inducible transcription factors, consisting of three members: PPAR-α, PPAR-β, and PPAR-γ. The PPAR signaling pathway plays a critical role in the maintenance of metabolic homeostasis, lipid and glucose metabolism, adipogenesis, and inflammation [22], and has been targeted in the development of drugs for the treatment of MAFLD, such as PPARγ agonist pioglitazone [23]. In a randomized control study, pioglitazone was found to reduce the serum aminotransferase levels, hepatic steatosis, and lobular inflammation [24]. A recent study showed that pemafibrate, a selective PPARα agonist, could prevent MASH development, although no reduction was seen in the hepatic triglyceride content [25]. However, a study conducted by Francque et al. [26] showed that the gene expression of PPARα in NASH patients is negatively correlated with the severity of steatosis, ballooning, and fibrosis. Such seemingly contradictory results indicate that possibly PPARs play different roles in different stages of MAFLD, or that the results at the protein level were not always consistent with those at the transcriptional level.

In the present study, we identified four interesting potential candidate proteins that might promote MAFLD development: EHHADH, ACOX1, ACOT4, and PEX11A. Of these, EHHADH was higher ranked in terms of the MCC algorithm, and we further validated it using RT-qPCR, GEO dataset, western blotting and immunohistochemistry. We believe that further studies should focus on the function of EHHADH in the progression of MAFLD. It is worth noting that all these proteins are closely associated to the PPAR signaling pathway. EHHADH protein, encoded by *Ehhadh* gene, is a bifunctional enzyme that is one of the four enzymes involved in the classical peroxisomal fatty acid beta-oxidation pathway [27]. Limited studies have focused on the role of EHHADH protein in the progression of MAFLD. A previous study conducted by Banasik et al. [28] also identified *Ehhadh* as a hub gene for MAFLD using a bioinformatics approach. EHHADH could regulate the expression levels of PPARα according to a given metabolic condition by binding to PPARα, thus influencing MAFLD progression. ACOX1, a target protein of PPARα, is the rate-limiting enzyme of fatty acid oxidation and could be used as an indicator for mitochondrial oxidation activity [29]. The upregulated expression of *Acox1* gene results in increased fatty acid oxidation in the mitochondria. However, previous in vitro studies [30, 31] found the expression levels of *Acox1* to be downregulated in palmitate-treated HepG2 cells. The possible reason for this could be that the expression levels of *Acox1* are different at different stages of MAFLD, while the in vitro cell models do not represent all stages of MAFLD.

In addition, we also performed GO analysis to explore the functions of the DEPs. As expected, several GO terms including lipid metabolic process, cellular lipid metabolic process, fatty acid metabolic process, and very long-chain fatty acid metabolic process were found to be significantly enriched. Of note, most of these DEPs were associated with processes of lipid metabolism, indicating that lipid metabolism in the hepatocytes plays a critical role in the process of MAFLD development. Furthermore, KEGG analysis also identified some other signal pathways, such as biosynthesis of unsaturated fatty acids, steroid hormone biosynthesis, retinol metabolism, etc. These pathways might also contribute to development of MAFLD and could be focused upon in the future studies.

In clinical practice, ultrasonography is commonly used to diagnose MAFLD, but liver biopsy remains the ‘gold standard’ for identifying the presence of NASH, as no specific biomarker for NASH diagnosis. Our results suggest that these hub proteins may act as a panel to identify NASH from MAFLD patients. In addition, as there are no approved drugs for treating NASH currently, targeting the hub proteins or pathways might develop potentially new therapeutics for NASH.

Despite the above findings, there are still some limitations in the present study. First, bioinformatics analysis demonstrated only an association between the identified proteins and the phenotype of MAFLD. Further studies are needed to elucidate the exact molecular mechanism of these proteins in the development of MAFLD. Second, TMT-based proteomics technology yielded only ~ 4000 proteins in our study; In addition, only 71 DEPs were identified between the HFD and control groups when the fold-change threshold was set as > 1.5 or < 0.67. These numbers were found to be insufficient for proteomics profile analysis. Although a lower fold-change threshold could have yielded more DEPs, we chose fold change of > 1.5 or < 0.67 to enable more convincible results. Third, although only six mouse liver samples (three from each group) were included in the TMT-based proteomics analysis, these samples, different from human liver samples, had high homogeneity and reliability and were sufficient to detect the DEPs between the two groups.

## Conclusion

Our study provides a comprehensive analysis of protein expression patterns involved in the development of MAFLD. These results might provide new insights into understanding the mechanism of MAFLD progression, and help in the identification of potential targets for treatment of MAFLD.

## Supplementary information

**Additional file 1**. Primers used for RT-qPCR.

**Additional file 2**. Detailed information about the identified proteins using TMT-based proteomics technology.

**Additional file 3**. GO and KEGG analysis of 666 DEPs with* p* value < 0.05.

**Additional file 4**. Protein-protein interaction network of 666 DEPs.

**Additional file 5**. Protein-protein interaction network of identified 20 hub proteins from 666 DEPs.

## Data Availability

All data generated or analyzed during this study are included in this published article (and Additional files).

## Abbreviations

MAFLD: Metabolic associated fatty liver disease
TMT: Tandem Mass Tag
HFD: High fat diet
ND: Normal diet
DEPs: Differentially expressed proteins
RT-qPCR: Real Time Quantitative Polymerase Chain Reaction
KEGG: Kyoto Encyclopedia of Genes and Genomes
MASH: Metabolic associated steatohepatitis
PPAR: Peroxisome proliferator-activated receptor
