# Supplementary material for: Tandem Mass Tag-based quantitative proteomics analysis of metabolic associated fatty liver disease induced by high fat diet in mice
Source: Nutr Metab (Lond). 2020 Nov 18;17:97. doi: 10.1186/s12986-020-00522-3 (PMC7672977; doi:10.1186/s12986-020-00522-3)
Supplement: Supplementary file 3 — Additional file 3. GO and KEGG analysis of 666 DEPs with p value < 0.05. [file 12986_2020_522_MOESM3_ESM.pdf]

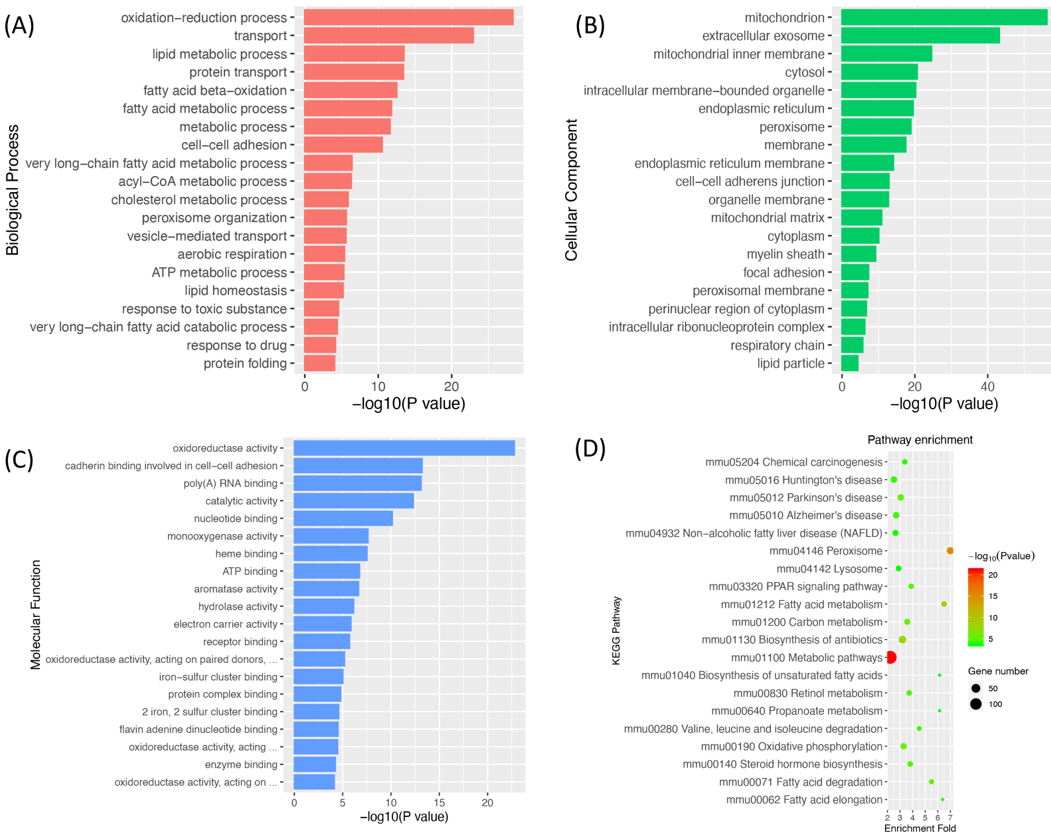

Supplemental file 3: GO and KEGG analysis of 666 DEPs with p value<0.05. DEPs are classified into biological process (A), cellular component (B) and molecular function (C). (D) KEGG pathway enrichment analysis of 666 DEPs. Size of circles indicates the gene number. Color of the circles represents -log10(P value)
