# Supplementary material for: Tandem Mass Tag-based quantitative proteomics analysis of metabolic associated fatty liver disease induced by high fat diet in mice
Source: Nutr Metab (Lond). 2020 Nov 18;17:97. doi: 10.1186/s12986-020-00522-3 (PMC7672977; doi:10.1186/s12986-020-00522-3)
Supplement: Supplementary file 4 — Additional file 4. Protein-protein interaction network of 666 DEPs. [file 12986_2020_522_MOESM4_ESM.pdf]

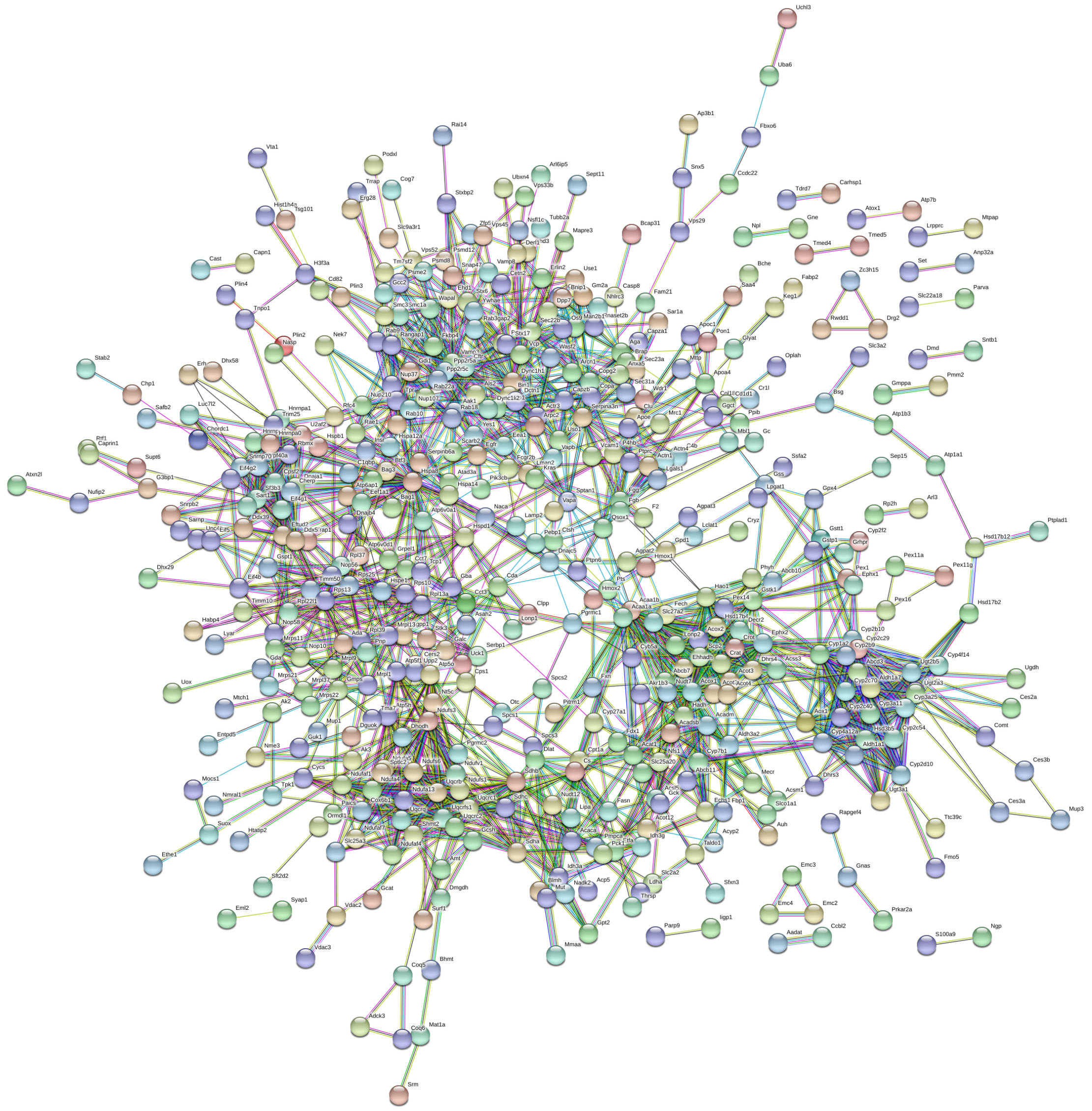

Supplemental file 4: Protein-protein interaction network of 666 differentially expressed proteins
